# Stemness signature and targeted therapeutic drugs identification for Triple Negative Breast Cancer

**DOI:** 10.1038/s41597-023-02709-8

**Published:** 2023-11-20

**Authors:** Samina Gul, Jianyu Pang, Hongjun Yuan, Yongzhi Chen, Qian yu, Hui Wang, Wenru Tang

**Affiliations:** Laboratory of Molecular Genetics of Aging & Tumor, Medical School, Kunming University of Science and Technology, 727 jingming south road, Kunming city, Yunnan province 650500 China

**Keywords:** Data mining, Breast cancer, Cancer stem cells

## Abstract

Triple-negative breast cancer (TNBC) is the most aggressive subtype of breast cancer and carries the worst prognosis, characterized by the lack of progesterone, estrogen, and HER2 gene expression. This study aimed to analyze cancer stemness-related gene signature to determine patients’ risk stratification and prognosis feature with TNBC. Here one-class logistic regression (OCLR) algorithm was applied to compute the stemness index of TNBC patients. Cox and LASSO regression analysis was performed on stemness-index related genes to establish 16 genes-based prognostic signature, and their predictive performance was verified in TCGA and METABERIC merged data cohort. We diagnosed the expression level of prognostic genes signature in the tumor immune microenvironment, analyzed the TNBC scRNA-seq GSE176078 dataset, and further validated the expression level of prognostic genes using the HPA database. Finally, the small molecular compounds targeted at the anti-tumor effect of predictive genes were screened by molecular docking; this novel stemness-based prognostic genes signature study could facilitate the prognosis of patients with TNBC and thus provide a feasible therapeutic target for TNBC.

## Introduction

Breast cancer has placed second on the list of common diseases worldwide, according to the World Health Organization (WHO) reports^1^. Every year women are diagnosed with approximately 268,600 new cases of invasive breast cancer, and about 41,760 women will die from breast cancer estimated by the American Cancer Society. Breast cancer is the sixth leading cause of cancer-related deaths among Chinese women, and approximately 11% of all breast cancers worldwide occur in China^2^. Breast cancer is among one of the most common cancer and, in China, is approximately more than twice the global incidence rate and is the sixth leading cause of cancer-related death^3^. TNBC triple-negative breast cancer is regarded as aggressive among all the subtypes. It lacks the expression of estrogen receptor, progesterone receptor, and human epidermal growth receptor2 and has an elevated risk of recurrence, metastasis, and higher histologic grade compared to other subtypes^4^. Patients with TNBC, when compared with patients with hormone receptor (HR)-positive breast cancer, experienced a dramatic increase in death within two years of diagnosis and worse overall survival, according to the data presented to National Comprehensive Cancer Network centers (NCCN) in 2012^5^. Each year approximately 15–20% of the more than one million breast cancer patients with TNBC are diagnosed worldwide^6^. No targeted therapies are available for TNBCs, unlike endocrine therapy for PR+ ER+ and HER2+ patients. Therefore, to improve the survival rate of TNBC, patient detection of potential markers and therapeutic targets need to be explored.

There is growing evidence that cancer stem cells have been studied in many solid tumors, including lung cancer^7^, ovarian cancer^8^, Hepatic carcinoma^9–11^, and pancreatic carcinoma^11^, colon^7^, and play an essential role in different human malignancies. Cancer stems cell research has revealed the unique function of cancer stem cells defines a specific cell type that possesses the main properties of self-renewal, differentiation potential, multi-lineage, and proliferation. The term stemness refers to the degree to which cancer stem cell contains these functional properties^12^. Accumulated evidence has also found that cancer stem cells play an essential role in cancer metastasis, differentiation^13^, and elimination of cancer stem cells will suppress the growth and recurrence of breast cancer^14^. Therefore, investigating the cancer stem cell in TNBC may improve the clinical results. The identification of reliable tumor markers will significantly impact TNBC prognosis and treatment. Cancer stem cells are a robust heterogeneous population and the cellular sources of unlimited growth and recurrence of malignant tumors. Cancer stem cells play critical roles in breast cancer growth, metastasis, and drug resistance^15^. The advanced understanding of the molecular mechanisms of CSCs in TNBC provides suitable disease management in the future. Accumulated evidence has revealed mRNAsi-related signature in different cancers, including lung squamous cell carcinoma, glioma, hepatocellular carcinoma, triple-negative breast cancer, and colorectal cancer^16^. However, there are few studies on the stemness index in TNBC. Therefore, findings of the stemness index’s application value in TNBC are critical to improving diagnosis and treatment.

The messenger RNA (mRNA) expression-based stemness index (mRNAsi) is used to quantify the unique characteristics of CSCs; Malta *et al*. developed a scoring system using one class logistic regression (OCLR) machine learning algorithm as a robust method to quantify the cancer stemness^17^. Here in this work, we explored the role of stemness index in 127 patients with TNBC to calculate the mRNAsi of TNBC samples using one class logistic regression algorithm, counted the stemness index and immune score for 127 TNBC samples, and analyzed the association between immune infiltration and mRNAsi. Then, we identified the stemness-related DEG into high and low mRNAsi groups and performed functional enrichment analysis to reveal the potential functions of these genes in the progression and pathogenesis of TNBC. Then, we classified the TNBC patients into two stemness subtypes using the consensus clustering method based on these DEGs. A novel prognostic risk model including sixteen genes (BMP4, CCBE1, CELSR3, CT83, CXCL11, EGR2, GLDC, GPRC5C, TRO, STMN2, SCGB2A2, RUNDC3B, PROS1, PCDHGA3, IL1RL1, UGT2B11) was established by COX and LASSO regression analysis, and its predictive performance was verified in external validation cohort. We constructed a nomogram for patients with TNBC for potential clinical application. We analyzed the TIME map for prognostic genes, analyzed single-cell RNA sequencing (scRNA-seq) data and explored (TIME) the tumor immune microenvironment, analyzed the fate of cells, and explored the expression of sixteen genes in different cell types. Besides, we further validated the expression levels of prognostic genes using the HPA database. Finally, molecular docking research was performed on sixteen genes to screen anti-stemness compounds.

## Results

### Correlation between mRNAsi and clinical characteristics of TNBC patients

**Fig. 1:**
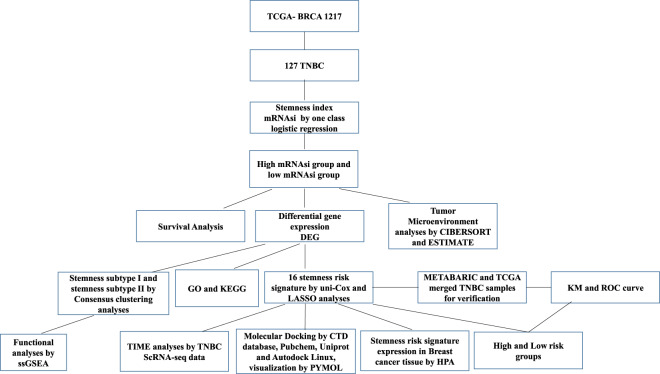
Work flow of current work.

**Fig. 2:**
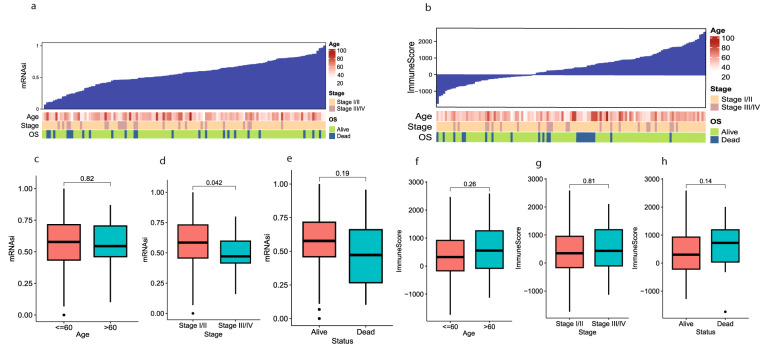
The correlation between mRNAsi and clinical characteristics in TNBC patients (N = 127). (**a**) The general diagram of the association between mRNAsi and the clinical features and mRNAsi score were shown on the y-axis (**b**) The general diagram of the association between the immune score and the clinical features, immune score were shown on the y-axis (**c**–**e**) The Boxplots of mRNAsi score for TNBC patients stratified by clinical features. (**f**–**h**) The boxplot of the immune score and clinical features of TNBC patients. Significance P values were calculated by the Wilcoxon rank sum test.

This work was performed according to the flow chart, which presents the overall construction scheme of the stemness index and stemness prognostic signature displayed in (Fig. 1). To explore the correlation between mRNAsi and clinical characteristics of TNBC, we calculated the stemness index and immune score of 127 TNBC patients using the OCLR and ESTIMATE algorithms. We then ranked patients to investigate the relationship between mRNAsi and clinical characteristics (Fig. 2a,b). We divided all patients into groups and then compared the mRNAsi expression in various clinical features according to the clinical characteristics. Association analysis showed that mRNAsi did not significantly differ by age (Fig. 2c), and mRNAsi were significantly high in clinical stage I/II (*p* = 0.042) (Fig. 2d). We found that the value of mRNAsi in the survival group was insignificantly higher than that in the group that died (Fig. 2e). There were no significant differences in the immune scores by age, clinical stage, or survival groups (Fig. 2f–h).

### Correlation, differential and functional enrichment analysis between mRNAsi groups and tumor microenvironment

**Fig. 3:**
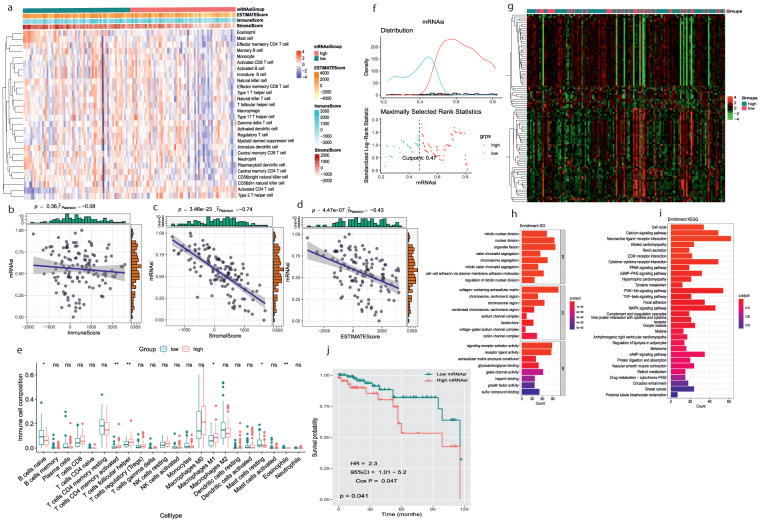
Correlation of mRNAsi with TIME different patterns of TNBC patients, different functional enrichment analyses, and survival outcomes between mRNAsi groups. (**a**) Based on the ssGSEA immune signature the mRNAsi of TNBC patients were categorized into two subgroups low and high group based. Red color indicates a high mRNAsi group and green color indicates a low mRNAsi group, the expression of an estimate, stromal, and immune, mRNAsi groups in the immune signature were indicated by red and blue color, red shows upregulation, and blue shows downregulation (**b**–**d**) Correlation between mRNAsi, the immune score, stromal score, and ESTIMATE score based on ESTIMATE algorithm. The blue line is the regression line of mRNAsi and other scores. (**e**) Comparisons of the abundances of 22 immune cells in 2 mRNAsi groups by CIBERSORT algorithm. *p < 0.05; **p < 0.01; ***p < 0.001, ****p < 0.0001. (**f**) mRNAsi were divided into two groups, high and low based on survminer analysis determined 0.47 as the optimal grouping, with value of high mRNAsi group (N = 85) and low mRNAsi group (N = 42). (**g**) The heatmap reflects the expression levels of DEGs between high and low mRNAsi groups. (**h-i**) The GO and KEGG functional annotation analysis of the DEGs. (**j**) KM survival analysis showed the different survival status between mRNAsi groups green color shows low mRNAsi and the red color shows high mRNAsi group with a p-value of 0.041. Significance P values were calculated by the Wilcoxon rank sum test.

To explore the correlation between mRNAsi and immune infiltration, we applied the ssGSEA method to quantify the enrichment of 28 immune-related signatures to reflect the immune activity. The result showed that in the low-mRNAsi group, the immune activity was higher than that in the high-mRNAsi group (Fig. 3a). ESTIMATE and CIBERSORT algorithms explored the tumor microenvironment, and we found that mRNAsi was significantly negatively correlated with the immune score, stromal score, and ESTIMATE score (*p* < 0.01), which indicated that the immune cell infiltration levels decrease with elevated TNBC stemness (Fig. 3b–d). We also detected the immune infiltration using the CIBERSORT algorithm to quantify the abundances of the 22 immune cell types in the two mRNAsi groups. We found that the mRNAsi was significantly positively correlated with T cell follicular helper cells, T cell CD4 memory activated, and M1 macrophages and was significantly negatively correlated with naive B cells, mast cells resting, and Eosinophil (Fig. 3e). There were no significant differences between samples grouped by the median mRNAsi value to explore the differences in the functional annotation and pathway enrichment analysis between the groups categorized by mRNAsi. For more reasonable grouping, we reclassified 127 TNBC patients into the high-mRNAsi group (*n* = 85) or the low-mRNAsi group (*n* = 42). We obtained an optimal cutoff of mRNAsi = 0.47 based on the results of the “survminer” analysis (Fig. 3f). We then analyzed the differential expression and identified 2228 DEGs from the intersection of mRNAsi groups and TNBC (Fig. 3g). To investigate the possible biological functions of these DEGs, we performed DAVID. According to the results of the functional enrichment analysis, we found mitotic nuclear division, regulation of mitotic nuclear division, cell−cell adhesion via plasma−membrane adhesion molecules, mitotic sister chromatid segregation, chromosome segregation, sister chromatid segregation, organelle fission nuclear division enriched biological processes. Cellular components, including chromosomal region, chromosome centromeric region, collagen−containing extracellular matrix and molecular functions, including glycosaminoglycan binding, extracellular matrix structural constituent, receptor-ligand activity, signaling receptor activator activity (Fig. 3h); and 30 enriched KEGG pathways, including the PI3K-Akt signaling pathway, TGF-β signaling pathway, MAPK signaling pathway (Fig. 3i). These results suggest that these DEGs are associated with the cancer signaling pathway and may regulate cancer progression. To check whether the mRNAsi significantly related to overall survival in TNBC, we conducted the K-M analysis, and the results showed that the patients with high mRNAsi scores showed poor overall survival status (Fig. 3j).

### Identification of TNBC stemness subtypes and exploration of tumor microenvironment

**Fig. 4:**
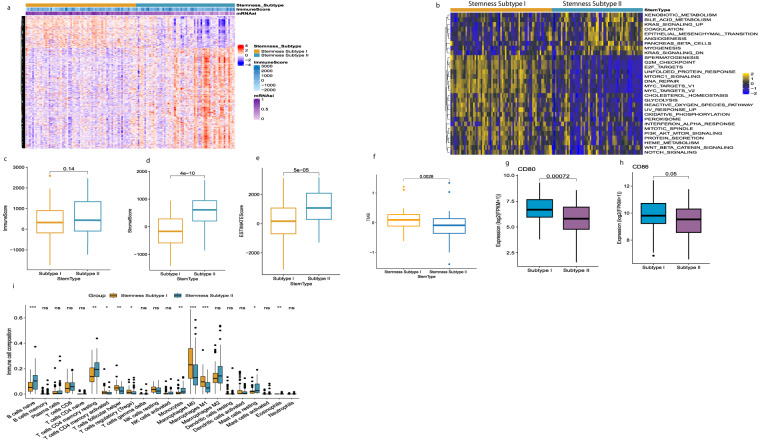
Identification of the two stemness subtypes, different TIME pattern, TMB, and immune checkpoint patterns between stemness subtype I shown by yellow color (N = 67) and stemness subtype II shown by blue color (N = 60) (**a**) The heatmap of the expression pattern of DEGs between the two stemness subtypes, immune scores, and mRNAsi groups. Red color indicates upregulation and blue color indicates downregulation (**b**). GSVA heatmap showed 33 differentially enriched pathways between the 2 stemness subtypes. Yellow color indicates upregulation and blue color indicates downregulation (**c**–**e**) Comparisons of the immune score, stromal score, and ESTIMATE score in stemness subtype I and II by ESTIMATE algorithm. Significance P values were calculated using Student’s T. test (**f**) TMB difference between the stemness subtypes I (N = 67) and II (N = 60). Significance P values were calculated by the Wilcoxon rank sum test. (**g-h**) The expression levels of CD86 and CD80 in the stemness subtypes I (N = 60) are shown by blue color and II by purple color (N = 60). (**i**) Comparisons of the abundances of 22 immune cells in the stemness subtypes I (N = 60) and II (N = 60) by CIBERSORT algorithm *p < 0.05; **p < 0.01; ***p < 0.001, ****p < 0.0001.

We utilized an unsupervised consensus clustering method to construct a novel classification of TNBC in the TCGA cohort to analyze the association between mRNAsi and TNBC subtypes. Therefore, 127 patients with TNBC were classified into two stemness subgroups (Fig. 4a), including stemness subtype I (67 patients, 53.7%) and stemness subtype II (60 patients, 47.2%). The demographic information between the two subtypes is shown in (Supplementary Table S1); according to the consensus heatmap and consensus CDF curve, the intergroup connections were the lowest, and the intragroup connections were the highest when k = 2 (Supplementary Fig. 1a,b; and Supplementary Table S2). We performed differential expression analysis in stemness subtypes and explored the molecular pathways associated with the stemness subtypes using GSVA. Finally, we identified 30 significantly enriched pathways positively related to the stemness subtype I (Fig. 4b). The results revealed that stemness subtype I tumors were primarily associated with tumorigenesis NOTCH-signaling, PI3K_AKT_MTOR-signaling, and WNT_BETA_CATENIN- signaling. We utilized ESTIMATE and CIBERSORT algorithms to elucidate different immune infiltration in two different stemness subtypes, as shown in (Fig. 4c–e); stromal score, immune score, and ESTIMATE score all appeared to be lower in stemness subtype I compared with stemness subtype II. Somatic mutation analysis revealed that TMB was significantly higher in the stemness subtype I (*p* = 0.0028, as shown in Fig. 4f). In general, the more efficacious treatment with an immune checkpoint inhibitor possible with the higher the TMB. Subsequently, CIBERSORT illustrates the immune cell infiltration abundances in TNBC. Immune infiltration including B cell naïve, CD4+ cell subsets, monocytes, and Mast cells significantly more enriched in stemness subtype II and CD4+ memory activated cell, T cell follicular helper, T cell regulatory, Macrophages M0, Macrophages M1 significantly more enriched in stemness subtype I (Fig. 4i). We also explored the expression level of six immune checkpoint genes, including (CD80, CD84, CD274, CTLA4, PDCD1, and PDCD1LG2) in stemness subtypes. We found that the stemness subtype I have a higher immune checkpoint expression level than stemness subtype II, as shown in (Supplementary Fig. 2a–d) but only significant in CD80, CD84 (Fig. 4g,h). These results suggest that stemness subtypes show different responses to immunotherapy and stemness subtype I am more immunogenic than stemness subtype II, and patients in stemness subtype I might show a better response to immunotherapy.

### Construction and validation of stemness related to an independent prognostic signature for TNBC

**Fig. 5:**
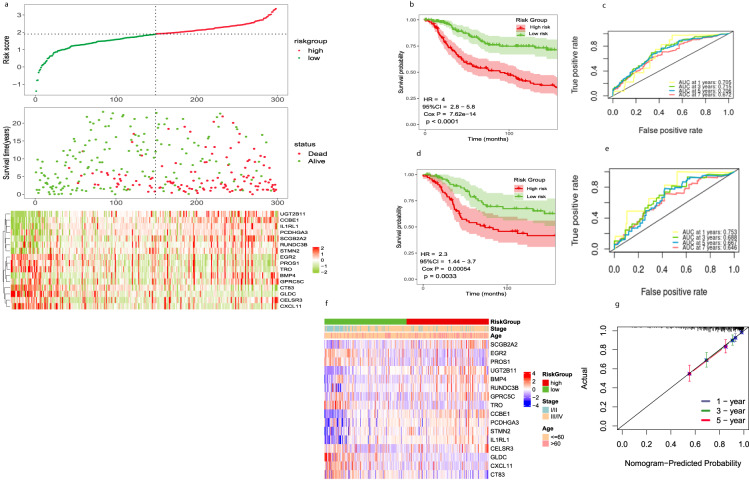
Construction and validation of an independent prognostic signature. (**a**) Risk score distribution (the high-risk group was shown by red color and low risk was shown by green color), survival status (dead were shown by red color and green indicates alive), and signature gene expression in the TCGA (N = 127) and METABRIC (N = 320) merged data cohort (N = 447) divided into training cohort (N = 298) and testing cohort (N = 149) red color shows upregulation and green shows downregulation. (**b**) The KM curves of the merged training cohort (N = 298) were divided into a low-risk group have green color (N = 149) and a high-risk group have red color (N = 149). (**c**) The ROC curve of merged training cohort, low (N = 149), high (N = 149) risk group. 1-year area under was shown by yellow color, 3 years by green color, 5 years by blue and 7 years by red color. (**d**) The KM curve of the merged testing cohort (N = 147) was divided into a low-risk group (N = 75) and a high-risk group (N = 74). (**e**) The ROC curve of the merged testing cohort is low (N = 75) and high (N = 74) risk group. (**f**) The heatmap for the connections between the risk groups and clinical characteristics and sixteen genes upregulation were shown by red color and downregulation were shown by green color (**g**) The Nomogram calibration curves to predict the 1-, 3-,  and 5-year survival. 1 year represented by blue color, 3 years by green color, and 5 years by red color.

We constructed a mRNAsi-related prognostic signature based on 2228 DEGs to predict TNBC prognosis. We used METABERIC and TCGA-BRCA merged datasets and extracted 447 TNBC samples, split into Train (sample; 298) and Test (sample; 149). We trained 2228 DEGs on 298 TNBC samples as a training cohort and identified 47 genes related to TNBC prognosis (*p* < 0.05) using univariate Cox regression analysis. We reduced this number to 16 genes using LASSO regression analysis (Supplementary Fig. 3a,b). Then, we used these 16 genes including (BMP4, CCBE1, CELSR3, CT83, CXCL11, EGR2, GLDC, GPRC5C, TRO, STMN2, SCGB2A2, RUNDC3B, PROS1, PCDHGA3, IL1RL1, UGT2B11) build prognosis risk signature. Among them, CT83, CXCL11, EGR2, GLDC, PROS1, TRO, and UGT2B11 were associated with decreased risk with HR < 1, while the other genes were related to an increased risk with HR > 1. The prognostic formula to calculate the risk score of each sample is as follows: Risk score = Coef (genes) × Exp (genes); Exp represents the gene expression level, and Coef represents the LASSO coefficient of the target gene. The risk scores of TNBC patients in 298 training datasets and 149 test datasets as validation cohorts were calculated, and the optimal cutoff values of the risk score in the training and test datasets were 1.7 and 1.8, respectively. TNBC patients in the Train dataset were divided into high-risk (n = 149) and low-risk (n = 149) groups according to the median value. The association between the risk score and survival information is exhibited in (Fig. 5a). In the Train cohort, the patients in the low-risk group had significantly longer overall survival times (*p* < 0.0001, HR = 4, 95%CI: 2.8 − 5.8; Fig. 5b). The AUC was 0.705 for the 1-year survival, 0.715 for the 3-year survival, 0.706 for the 5-year survival, and 0.672 for the 7-year survival (Fig. 5c), indicating that the signature has high precision. We used the same method to assign 149 test cohorts to low-risk groups (*n* = 75) or high-risk groups (*n* = 74). In the test cohort, patients in the low-risk group had lower death rates and longer survival times (*p* = 0.0033, HR = 2.3, 95% CI: 1.44–3.7; Fig. 5d). The AUC of the test cohort, 0.753 for the 1-year survival, 0.688 for the 3-year survival, 0.667 for the 5-year survival, and 0.646 for the 7-year survival, also indicated that the model has predictive power (Fig. 5e). We used Cox regression analysis to check the independent predictive ability of the TNBC prognostic model. The univariate Cox analysis demonstrated that the risk score and age were prognostic factors (*p* = 7.62e−14, HR = 4.039, 95%CI: 2.801−5.824), and the multivariate Cox analysis demonstrated that the risk score was an independent factor for TNBC (*p* = 6.25e−12, HR = 3.810, 95%CI: 2.602−5.579; Supplementary Fig. 4a,b). We plotted the expression level of sixteen genes between the two risk subgroups (Fig. 5f) and found that ten genes were highly expressed in the high-risk group, suggesting that they may regulate TNBC progression. We used the prognostic signature to establish a nomogram (Supplementary Fig. 5a). The calibration curves were used to compare the actual probabilities of survival and predicated survival rates for the 1-year, 3-year, and 5-year survival, indicating a significant correlation between the actual survival rate and the survival rate predicted by the nomogram (Fig. 5g). This suggests that the nomogram has a great predictive value in the prognosis of patients with TNBC.

### Small molecular compounds docking of prognostic genes

**Fig. 6:**
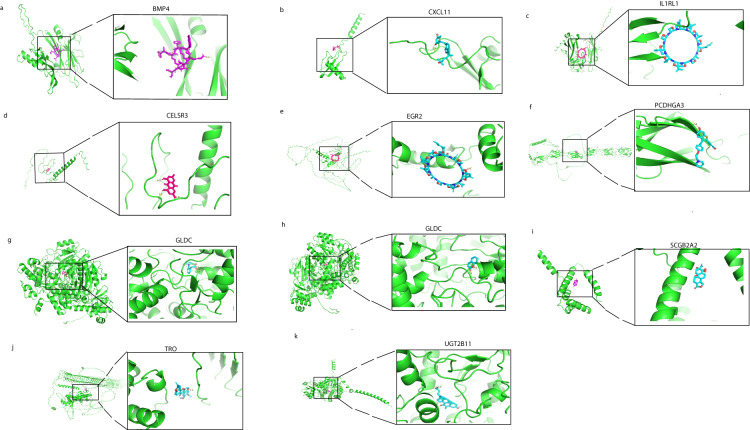
The docking results of proteins encoded by prognostic genes with small molecular compounds. The docking results of BMP4 with Octreotide (**a)**. The docking results of CXCL11 with Calcitriol (**b**). The docking results of ILIRL1with Cyclosporine (**c**).The docking results of CELSR3 with Emodin (**d**). The docking results of EGR2 with Cyclosporine (**e**). The docking results of GLDC with Abrine (**g**) and Coumarin (**h**).The docking results of PCDHGA3 with Raloxifene Hydrochloride (**f**). The docking results of SCGB2A2 with Triptonide (**i**). The docking results of TRO with Rotenone (**j**). The docking results of UGT2B11 with Obeticholic acid (**k**).

In this work, we identify drugs targeted to prognostic genes using screening of the CTD database, Autodock molecular docking, and drug toxicology studies. Octreotide binds tightly to BMP4 (Fig. 6a), upregulates BMP4 mRNA expression, and their simulated binding energy for molecular docking was −8.32 (kcal/mol). The results of the molecular docking analysis indicated Calcitriol binds tightly to CXCL11 (Fig. 6b) and regulates the mRNA expression with binding energy for molecular docking was −11.47 (kcal/mol). Cyclosporine stood out with an optimal docking binding energy of – 11.03 (kcal/mol) with an increase in mRNA expression of ILIRL1 (Fig. 6c). Emodin decreases mRNA expression of CELSR3 with −5.94 (kcal/mol) binding energy (Fig. 6d). Among the small molecule compound, Cyclosporine stood out with optimal binding energy of – 62.3 (kcal/mol) and increased EGR2 mRNA expression (Fig. 6e). Raloxifene Hydrochloride efficiently increases mRNA expression of PCDHGA3 with a simulated binding energy of −7.22 (kcal/mol) (Fig. 6f). Abrine and Coumarin stood out with an optimal docking binding energy of − 6.55 and −6.5 (kcal/mol) with an increase in mRNA expression of GLDC, respectively (Fig. 6g,h). Triptonide enhanced mRNA expression of SCGB2A2 with optimal docking energy was −9.17 (kcal/mol) (Fig. 6i). Rotenone exhibited a docking binding energy of −6.37 (kcal/mol), enhancing the mRNA expression of TRO (Fig. 6j). Finally, in screening for small molecule compounds that upregulate UGT2B11 mRNA, obeticholic acid molecular docking binding energy was − 7.99 (kcal/mol) (Fig. 6k). In summary, we have selected ten small molecular compounds that are beneficial for improving the worse prognosis caused by sixteen genes, providing new research ideas for targeted therapy of TNBC.

### Exploration of the mRNA and protein expression levels of sixteen signature genes

To explore the clinical significance of the 16 stemness-related genes in the model used clinical specimens from the HPA database, HPA analysis showed that the protein levels CCBE1, CELSR3, CXCL11, GLDC, GPRC5C, PROS1, PCDHGA3, RUNDC3B were shown in Breast cancer tissues compared to normal Breast tissue and BMP4, CT83, TRO, STMN2, SCGB2A2, EGR2, IL1RL1, UGT2B11 were not found in database. It was discovered that to the TNBC stemness index, five genes related, including RUNDC3B, PROS1, PCDHGA3, CCBE1, and GPRC5C, were downregulated in cancer tissue compared to normal Breast tissue, and GLDC, CXCL11, and CELSR3 were up-regulated in breast cancer tissue compared to normal tissue as shown in (Supplementary Fig. 6).

### Prognostic genes expression in TIME explored scRNA sequencing data

**Fig. 7:**
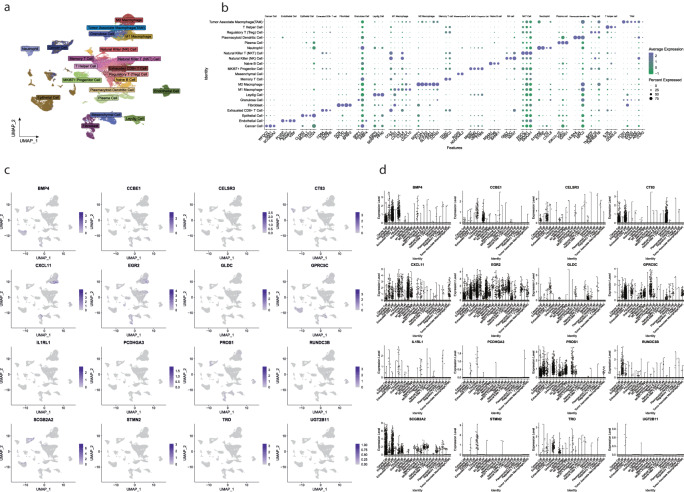
Single-cell analysis of 16 genes. (**a**) 21 different cell populations in the tumor immune microenvironment in patients with TNBC (N = 10) (**b**) Marker gene of 21 different cells. The purple color shows higher expression of marker genes and the green color shows low expression (**c**) Feature Plot of 16 genes in 21 cells blue color shows high expression and light blue shows low expression. (**d**) Vln Plot with 16 genes in 21 cells.

The tumor immune microenvironment of 10 patients with TNBC was resolved after single-cell RNA sequencing data were analyzed according to the standard workflow of Seurat. A total of 21 different types of cell clusters were annotated (Fig. 7a,b): Cancer Cell (WFDC2, SAA1, SCGB2A2), Endothelial Cell (PLVAP, ACKR1, VWF), Epithelial Cell (CLDN3, MUCL1, TFF3), Exhausted CD8 + T Cell (CD8A, CD8B, CXCR6), Fibroblast (DCN, APOD, LUM), Granulosa Cell (SPP1), Leydig Cell (ASPH, SERPINE1, PFN2), M1 Macrophage (CXCL9, CXCL10, CXCL2), M2 Macrophage (SEPP1, F13A1, FOLR2), Memory T Cell (TRAC, TRBC2), Mesenchymal Cell (RGS5, NDUFA4L2), MKI67+ Progenitor Cell (MKI67, RRM2, UBE2C), Naive B Cell (MS4A1, CD79B, BANK1), Natural Killer (NK) Cell (GNLY, TRDC, NKG7), Natural Killer T (NKT) Cell (RGCC, DNAJB1, DNAJA1), Neutrophil (S100A8, S100P, PI3), Plasma Cell (IGKV3-15, IGKC, IGHG1), Plasmacytoid Dendritic Cell (LILRA4, PLD4, NPC2), Regulatory T (Treg) Cell (BATF, TNFRSF18, TNFRSF1B), T Helper Cell (IL7R, CCR7, CD40LG), Tumor Associate Macrophage(TAM) (FCN1, VCAN, EREG).

**Fig. 8:**
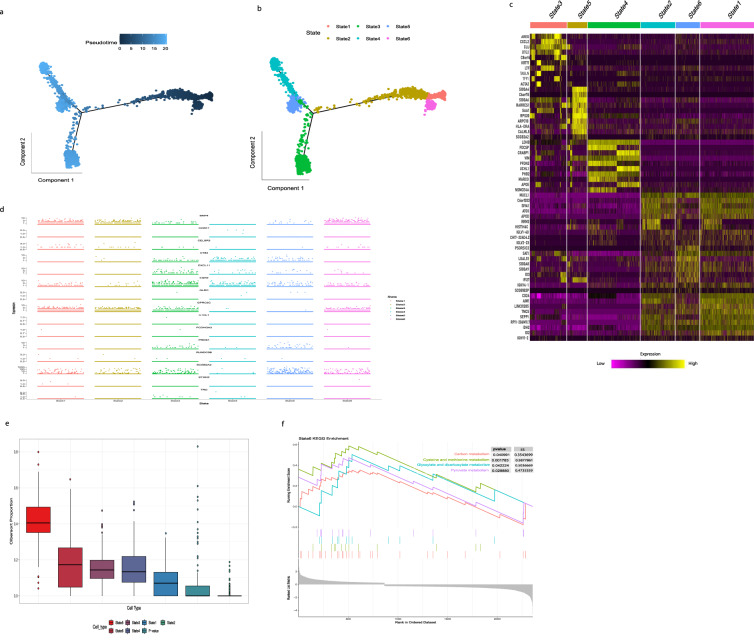
Pseudotime analysis of epithelial cells. (**a**) pseudotime of epithelial cell, (**b**) Epithelial cells divided into six differentiation states, state 1 was shown pink, state 2 was shown yellow, state 3 was shown dark green, state 4 by light green, state 5 blue, and state 6 light purple (**c**) Heatmap shows the top of 10 markers in six differentiation states, low expression of genes was shown purple and higher expression was shown yellow (**d**) Jitter plot showing sixteen genes expression in six differentiation states of epithelial cells (**e**) differentiation state six infiltration ratio queue by CIBERSORT algorithm (**f**) GSVA (KEGG term) analyzes state six of differentiation states, the right panel shows p-value and enrichment score.

Subsequently, the expression differences of 16 genes in 21 different immune cells were observed (Fig. 7c,d). BMP4 is highly expressed in Epithelial and Fibroblast cells, and CCBE1 shows high expression in Leydig Cells. CELSR3 is expressed in Epithelial Cells, CT83 is highly expressed in Cancer cells, Epithelial cells, and Neutrophils, and CXCL11 is expressed in Fibroblast and M1 Macrophages. EGR2 is highly expressed in Cancer, Epithelial Cell, Fibroblast, M1 Microphage, and Natural killer T cells. RUNDC3B is expressed in Epithelial Cells. GLDC is expressed in Neutrophils, and GPRC5C is highly expressed in Epithelial and Mesenchymal cells. PROS1 is highly expressed in Cancer Cells, Endothelial Cells, Fibroblasts, and M1 Macrophages, and; SCGB2A2 is highly expressed in Cancer Cells, Epithelial Cells, and Granulosa, TRO is expressed in Fibroblasts. IL1RL1, PCDHGA3, STMN2, and UGT2B11 showed no expression with tumor microenvironment components. As the target genes showed high expression in epithelial cells among all cells, we used Monocle for pseudotime trajectory analysis for Epithelial cells (Fig. 8a). The results showed that epithelial cells were divided into six Differentiation states (Fig. 8b) and the top of ten markers of each state (Fig. 8c); we checked the sixteen genes expression among six differentiation states of the epithelial cells (Fig. 8d) we found that state six showed higher infiltration among all the states by CIBERSORT algorithm (Fig. 8e) which may be related to tumor-promoting. GSEA analysis showed that State Six has significantly upregulated Glyoxylate and dicarboxylate metabolism, Cysteine and methionine metabolism, and Pyruvate metabolism (Fig. 8f), indicating that state six mainly participates in EMT epithelial-mesenchymal transition, mounting evidence showed that EMT acts as a driver during cancer metastasis in different cancer types.

## Discussion

According to recent studies, cancer stem cells play critical roles in cancer growth, metastasis, and therapy resistance^18,19^. It suggests the role of the cancer stemness index in TNBC should be further investigated. In this work, we used a series of bioinformatics algorithms to identify the TNBC stem cell-related prognostic gene signature, and we performed targeted drug screening of the prognostic genes signature to propose a therapeutic approach that regulates poor prognosis. In this research, we applied the OCLR machine-learning algorithm proposed by Malta *et al*. Combined with the PCBC dataset, calculated the mRNAsi score for each patient with TNBC. Based on the median mRNAsi value, we divided the patients into high and low mRNAsi groups. We found a notable negative correlation between mRNAsi and the TNBC immune score. The ssGSEA results showed that the low-mRNAsi group has significantly higher immune activity. This indicates that high mRNAsi is closely associated with a low abundance of immune cells, suggesting that CSCs may promote TNBC development by weakened immune cells’ abilities. We found that high mRNAsi have poor survival compared to low mRNAsi used KM analysis. We redefined the mRNAsi high and low groups based on survminer analysis and functional enrichment analysis. We found that DEGs are closely related to mitotic nuclear division, regulation of mitotic nuclear division, and enriched in cancer-promoting pathways, including the PI3K-Akt signaling pathway, TGF-β signaling pathway, MAPK signaling pathway, suggesting that the CSCs may regulate cancer progression. Using consensus clustering, we classified patients into two stemness subgroups, stemness subtype I and stemness subtype II. Stemness subtype II has higher enrichment of immune cells and immune infiltration compared to stemness subtype I. The expression level of CD80, CD86, and the TMB value was higher in subtype I compared to subtype II. This suggests that we could choose different clinical treatments based on stemness characteristics for patients with TNBC. In the present work, we identified 16 genes, including(BMP4, CCBE1, CELSR3, CT83, CXCL11, EGR2, GLDC, GPRC5C, TRO, STMN2, SCGB2A2, RUNDC3B, PROS1, PCDHGA3, IL1RL1, UGT2B11) related to TNBC stemness index prognostic signature and constructed a prognostic risk model. KM plot and a ROC curve indicated that the patients in the low-risk group had significantly longer overall survival times compared to the high-risk group. Furthermore, we constructed a nomogram for TNBC patients for potential clinical application. In this prognostic signature, STMN2, SCGB2A2, RUNDC3B, PCDHGA3, IL1RL1, BMP4, CCBE1, CELSR3, and GPRC5C were highly expressed in the high-risk group, and HR >1, suggesting the initiation and migration of TNBC.

Accumulated evidence suggests that 16 genes play a tumorigenic role in several cancers; BMP4 is vital in the progression of malignant melanoma, promotes melanoma cell invasion and migration, and acts as a tumor suppressor in breast cancer^20,21^. High expression of CCBE1, a novel potential biomarker to predict CRC patients’ prognosis, contributes to the aggressiveness and poor prognosis in Colorectal Cancer patients^22^. Xuefeng *et al*. verified CELSR3 as a potential biomarker for the prognosis of Hepatocellular carcinoma patients, with high expression of CELSR3 mRNA involved in cancer progression^23^. Chen Chen *et al*. verified that CT83 is the most specific gene for triple-negative breast cancer, and its high expression is associated with worse overall survival in breast cancer^24^. A high level of RBP-JK is significantly related to high CXCL11 expression, a risk factor for the poor overall survival of colon cancer patients verified by Mengjie *et al*. GLDC abnormal expression is observed in multiple cancer; its aberrant activation correlates with poorer survival in lung cancer patients^25^. Xueyan Zhang *et al*. verified that PCDHGA3 is associated with cell proliferation and expressed in Follicular lymphoma irrespective of B Cell Lymphoma2 status and grading^26^. PROS1 is a tumor-derived functional ligand for Tyro3 that protects cancer cells from acute apoptosis induced by staurosporine and supports cancer cell survival; it also acts as a tumor metastasis inhibitor^27,28^. EGR1/2 is involved in cell growth and apoptosis in different types of cancer and could inhibit tumor development, including Papillary Thyroid Carcinoma Cell Growth^29^. RUNDC3B, a methylation hotspot, may be a valuable biomarker for diagnosis and prognosis in lymphoid malignancies^30^. Iman *et al*. investigated SCGB2A2 immunostaining in bone marrow as a tool to investigate early Bone marrow micrometastases in breast cancer^31^. Mingrui Shao *et al*. identified that β-catenin/TCF mediated the transcription of STMN2, which promotes EMT and cell proliferation in pancreatic cancer. TRO plays an essential role in the development of Osteosarcoma and may be a significant potential biomarker and prognostic factor^32^.

The tumor microenvironment comprises fibroblasts, immune cells, endothelial cells, adipocytes, cytokines, and growth factors; the tumor immune microenvironment plays a key role in tumor growth, metastasis, therapeutic resistance, and maintenance of stemness^13,16^. Among 16 prognostic genes, CT83, CXCL11, EGR2, GLDC, PROS1, TRO, UGT2B11 were associated with low risk group with HR < 1 while BMP4, CCBE1, CELSR3, GPRC5C, STMN2, SCGB2A2, RUNDC3B, PCDHGA3, ILIRL1 were related to high risk group with HR > 1. We analyzed tumor immune microenvironment TIME in TNBC by analyzing the TNBC GEO scRNA-seq dataset. We found low-risk prognostic genes, including CT83, and GLDC, highly expressed in Neutrophils and CXCL11, and EGR2 showed high expression in M1 Microphages. Neutrophils and M1 macrophages play important roles in killing tumor cells; in cancer, the role of neutrophils is debated. Several studies showed that elevated numbers of neutrophils in the tumor are associated with poor prognosis; conversely, they inhibited tumor angiogenesis^33–35^. High-risk prognostic genes BMP4, CELSR3, GPRC5C, and RUNDC3B, highly expressed in Epithelial Cells, CCBE1 showed expression in Leydig cells, SCGB2A2 highly expressed in Epithelial Cells and Cancer cells, suggesting that Epithelial cell play an important role in tumor growth and initiation. We identified the Epithelial cells state with six distinct differentiation fates through developmental trajectory analysis; for further mining the heterogeneity of epithelial cells and exploring the sixteen genes expression among six states of differentiation, we found high infiltration ratio in state six and GSEA analysis showed that Glyoxylate and dicarboxylate metabolism, Cysteine and methionine metabolism, Pyruvate metabolism, significantly upregulated in state six, which play important in driving of EMT epithelial-mesenchymal transition, a driver during cancer metastasis in different cancer types showed that stemness genes promote EMT^36,37^.

In addition, the protein expression of all 16 genes in breast cancer was verified using the public HPA database. RUNDC3B, PROS1, PCDHGA3, CCBE1, and GPRC5C were found to be adversely linked with the TNBC stemness index and to be protective risk factors in the prognosis of TNBC on the other hand, GLDC, CXCL11, CELSR3 were upregulated, had a favorable link with stemness index.

In this work, we conducted targeted drug screening for 16 prognostic genes intending to propose a better therapeutic approach that regulates poor prognosis. The drugs we found out for our genes are FDA-approved drugs (Octreotide, Calcitriol, Cyclosporine, Emodin, Coumarin, Raloxifene Hydrochloride, Triptonide, Obeticholic acid). As a small molecule compound that can efficiently bind to BMP4 to upregulate the mRNA expression, Octreotide showed good performance in increasing the BMP4 expression; it is a synthetic somatostatin analog, a hormone with well-proven efficacy for the treatment of solid tumors, including breast, prostate, colon, pancreas, and small cell lung carcinoma^38^. The results of the molecular docking analysis indicated that Calcitrial bind tightly to CXCL11; Calcitriol, [1,25(OH)_2_D_3_] is the active hormonal form of vitamin D and regulates the balance of serum calcium and phosphate levels, which is essential for bone mineralization. If Calcitriol toxicity can be effectively managed, it exerts an anti‐osteosarcoma effect^39^. Cyclosporine showed good binding energy with EGR2, inhibiting intracellular Ca^2+^-mediated calcineurin phosphatase activity and inactivating the nuclear factor of activated T-cells (NFAT) pathway in immune cells and has the potential of being a therapeutic approach for the inactivation of NFATc1^40^. Abrine and Coumarin were found to have an affinity for GLDC. Abrine is an N (alpha)-methyl derivative of L-tryptophan, which improves the efficacy of immunotherapies by reducing the breakdown activity of tryptophan^41^. Benzopyrone is the basic structure of Coumarins, inhibits carbonic anhydrase, targets PI3K/AKT/MTOR signaling pathways, induces cell apoptosis protein activation, and inhibits tumor multidrug resistance and microtubule polymerization^42^. In the screened drug cohort, Raloxifene is a selective estrogen receptor modulator that binds to the estrogen receptor, induces autophagy via the activation of AMPK by sensing decreases in ATP, and promotes cell death in breast cancer cells^43^. Triptonide is a traditional Chinese herb that suppresses pancreatic cancer cell-mediated Tumor vasculogenic mimicry and inhibits the expression of VE-cadherin by reducing tumor cell migration and invasion^44^, showing good affinity towards SCGB2A2. Rotenone enhanced the mRNA expression of TRO, a toxic rotenoid compound that inhibited colon cancer cell proliferation, invasion, and migration through the PI3K/AKT pathway and promoted apoptosis^45^. Emodin down-regulates the mRNA expression of CELSR3, the primary chemical component of anthraquinone-induced apoptosis in cancer cells through cell cycle arrest^46^. Finally, in screening for small molecule compounds that upregulate UGT2B11 mRNA, obeticholic acid, the natural FXR agonist also known as INT-747 or 6α-ethyl-chenodeoxycholic acid inhibits Hepatocellular carcinoma proliferation, migration, and invasion via interfering with the activation of IL-6/STAT3 signaling pathway^47^. In this work, as mentioned above, sixteen prognostic genes targeting ten targeted drugs, our study has proposed a novel targeted therapy scheme consisting of a combination of multiple drugs, might would be better unless these drugs are validated in experiments or clinical trial.

At the same time, this study also had some limitations. First, we only included 10 scRNA-seq TNBC patients from GEO, a small sample size. Secondly, the two stemness subtypes showed apparent differences in immune infiltration, stromal, immune, and Estimate score were higher in steaminess subtype II and TMB, and immune checkpoint genes expression were more elevated in steaminess subtypes I; they may show different responses to immunotherapy. Therefore, it must be validated in future clinical experiments. The combined therapeutic value of these ten targeted drugs at the cellular and animal level will be the subject of future work. Our results are not experimentally validated, which is what future work will need. This research analyzed the association between mRNAsi and clinical characteristics and immune infiltration and identified two stemness-related molecular subtypes. We developed a stemness risk signature that can effectively predict the prognosis of patients with TNBC; we analyzed prognostic gene expression in normal and tumor tissue using immunohistochemistry and explored the predictive genes expression in TIME using the scRNA sequencing dataset and found that the high-risk stemness-associated genes promote EMT. Lastly, we screened drugs for the prognostic genes of risk signature, which led to new insights for targeted therapy.

## Methods

### TNBC data source and pre-processing

In the current study, the gene expression and corresponding clinical characteristic profiles of GDC TCGA Breast cancer (BRCA) with dataset ID TCGA-BRCA.htseq_counts.tsv with a total of 1217 samples were obtained from the University of California Santa Cruz (UCSC) Xena database (https://gdc.xenahubs.net) and converted the RNA-seq Counts to FPKM (fragments per kilobase of transcript per million mapped reads) and normalized by log2, extracted 127 samples of TNBC subtype of breast cancer for analysis. In this work, we extracted 320 samples of the TNBC subtype of breast cancer from METABRIC with gene expression data obtained from METABRIC (Breast Cancer) (http://www.cbioportal.org/datasets) with a total of 2509 samples, the log2 intensity value was already associated with HUGO gene symbol as Illumina probeset to HUGO gene symbol mapping was already done by cBioPortal and merged with 127 TNBC sample obtained from TCGA-BRCA and 447 patient data with TNBC were used as validation cohort. The stem cell expression profiles (syn2701943) were downloaded from the Progenitor Cell Biology Consortium database (https://www.synapse.org). The expression levels of 16 stemness-related genes were compared in Breast tumor tissues and normal tissues using Human Protein Atlas (HPA) database (https://www.proteinatlas.org/). GSE176078 has 10 TNBC samples downloaded from the GEO database (https://www.ncbi.nlm.nih.gov/geo/) and cell clusters were annotated with a Marker based on the SingleR package (v2.0.0) and the CellMarker database (http://117.50.127.228/CellMarker/). Somatic mutation data was downloaded from TCGA. For molecular docking we obtained data from three databases, we used CTD Database (https://ctdbase.org/) to download the catalog of small molecules that interacted with prognostic genes and then downloaded the small molecule structures from the PubChem Database (https://pubchem.ncbi.nlm.nih.gov/) Next, the Uniport Database (https://www.uniprot.org) was used to download the biological macromolecular structures translated by the prognostic genes.

### Calculation of stemness index for TNBC

In this work, we used the stem cell expression profiles (syn2701943) downloaded from the Progenitor Cell Biology Consortium database (https://www.synapse.org), and stemness signature were identified via one class logistic regression (OCLR) machine learning algorithm, and subsequent Spearman correlation was conducted between stemness hallmark and normalized 127 TNBC expression matrix to count the stemness index (mRNAsi) of each TNBC patient by scaling spearman correlation coefficients to be 0–1 accordingly; the higher the value, greater the tumor dedifferentiation and higher the activity of the cancer stem cells. According to the median mRNAsi, TNBC patients were placed into the high- and low-mRNAsi groups.

### Generation of differentially expression gene and functional enrichment analysis

Based on the two mRNAsi groups, the “limma” function was utilized to identify the DEGs between the high and low mRNAsi groups. The selection criteria for DEGs were an FDR < 0.05 and |log2 fold change (FC)| > 1. Gene Ontology (GO) was performed for functional annotation, and Kyoto Encyclopedia of Genes and Genomes (KEGG) were performed to assess related pathways, using the “ClusterProfiler” package for functional annotation.

### The exploration of (TME) tumor microenvironment infiltration and stemness index

We used Estimation of stromal and Immune cells in malignant Tumor tissues using Expression data (ESTIMATE) algorithm to characterize the TME obtained the immune scores (represent immune cell infiltration), stromal scores (symbolize abundance of stroma), and ESTIMATE scores (represents tumor purity) of TNBC patients via estimate R package, higher tumor purity, low degree of infiltration of immune cell in tumor and higher stemness index, based on median immune score TNBC patients were split into high- and low-immunity groups. We collected a set of 28 immune-related genes^48^ using the R package GSVA, performed a single sample Gene Set Enrichment Analysis (ssGSEA) to compute the rank value of each gene from the expression profile, and quantified the enrichment score of each gene in each sample can be used to determine the immune cell activity. CIBERSORT deconvolution algorithm^49^ was applied to quantify the relative abundance of 22 immune cells in a mixed population. The CIBERSORT method provides a set of gene signatures for 22 tumor-infiltrating immune cell fractions, including CD4+ resting memory T cell, memory B cell and naive B cell, etc.

### TNBC stemness subtypes and immune infiltration exploration

We applied an unsupervised Consensus clustering method to identify a novel stemness-based classification via the “ConsensusClusterPlus” R package. The clustering analysis was performed with 100 iterations, and 80% of sampling was used in each iteration. The consensus heatmap and cumulative distribution function CDF were visualized to select an optimal number of clusters and to explore the overall survival (OS) of different stemness subtypes Kaplan-Meier (K-M) curve was conducted. The gene set variation analysis (GSVA) was performed to explore Kyoto Encyclopedia of Genes and Genomes (KEGG) pathways in different stemness subtypes using the package “GSVA” R and the molecular signatures database (MSigDB) (http://www.gsea-msigdb.org/) was used to download the KEGG pathways profile. To explore the connection between immune infiltration and the stemness subtype, we compared the immune score and stemness subtype and the level of immune infiltration between different subtypes. Next, we compared immune checkpoints in different stemness subtypes, including PDCD1, CD80, CD274, PDCD1LG2, CTLA4, and CD86 expression levels. We also compared the differences in the tumor mutation burden (TMB) values between the different stemness subtypes using somatic mutation data downloaded from TCGA. We used R “maftools” to calculate the tumor mutation burden.

### Construction and validation of risk score model

Univariate Cox regression analysis was performed on DEG to identify the genes related to prognosis, and for subsequent analysis, the genes with significance were selected. LASSO, the least absolute shrinkage and selection operator regression analysis using the R package “glmnet,” was used to determine the meaningful genes in uni-Cox analysis to build the risk prognostic model using the regression coefficient and normalized expression value of the characteristic gene according to this formula as GeneExp1*Coef1 + GeneExp2*Coef2 + GeneExp3*Coef3 …. According to the formula the score was obtained, patients with TNBC were divided into the high-risk and low-risk groups by median values, the optimal cutoff values of the risk score in the training and validation datasets were 1.7 and 1.8, respectively, and to analyze overall survival in high-risk and low-risk groups, we plot Kaplan-Meier KM survival curve. We performed the receiver operating characteristics ROC curve drawn by the R package to verify the established model accuracy. METABERIC and TCGA merged data were used as a validation cohort.

### Exploration of prognostic factor and Nomogram construction

We evaluated clinical characteristics, including age and clinical stage, in combination with risk scores using Cox regression analysis to explore whether this risk model can independently prognosticate. Based on independent prognostic factors identified by Cox regression analysis, we constructed a prognostic nomogram and used calibration plots to test the predictive accuracy of the nomogram.

### Drug screened and docking

We screened fourteen protein-coding genes for targeted drugs based on functional studies of six teen prognostic genes. We used Autodock (Linux, v4.2) for molecular docking to study small molecule compounds interacting with prognostic genes. Firstly, we used CTD Database (https://ctdbase.org/) to download the catalog of small molecules that interacted with prognostic genes^50^ and then downloaded the small molecule structures from the PubChem Database (https://pubchem.ncbi.nlm.nih.gov/)^51^. Next, the Uniport Database (https://www.uniprot.org) was used to download the biological macromolecular structures translated by the prognostic genes^52^. Finally, the small molecule with substantial interaction with the biological macromolecules is determined by the lowest binding energy and is carried out according to the standard docking process. Moreover, PyMol (v2.6, Open-Source) visualizes the results.

### Detection of gene expression

The expression levels of 16 stemness-related genes were compared in Breast tumor tissues and normal tissues using clinical samples from the Human Protein Atlas (HPA) database (https://www.proteinatlas.org/) using “HPAanalyze” R package to retrieve detail of the 16 stemness genes from HPA, hpaXmlGet function was used to download the corresponding XML file for the desired gene, and hpaXmlTissueExpr function were used to extract the entire record of every staining available for both antibodies, including clinical data, original images, and staining quantifications.

### Exploration of prognostic genes in TIME using scRNA sequencing data

GSE176078 (*N*_*TNBC*_ = 10) was downloaded from the GEO database (https://www.ncbi.nlm.nih.gov/geo/) and used to analyze differences in expression levels of 16 genes at the single-cell level. Single-cell Count matrics were analyzed using a Seurat (v4.03) package using standard analysis tubes. The filtration standard of the mitochondrial genes was percent.mt < 15. The first 2000 Variable Features were used as the reference for data standardization. The first 20 PCs were taken as the inputs for UMAP nonlinear dimensionality reduction, and SNN cell clustering resolution = 0.4 was finally selected as the index for subsequent analysis. Subsequently, cell clusters were annotated with a Marker based on the SingleR package (v2.0.0) and the CellMarker database (http://117.50.127.228/CellMarker/). Pseudotime trajectories of epithelial cells were constructed using Monocle (v2.22.0), the algorithm uses machine learning techniques to use a specific set of genes as input to arranging the cells into trajectories with branch points, and the results are cell populations in different differentiation states, And perform functional Enrichment analysis of cells in different states.

### Statistical analysis

R version 4.1.1 was used for all statistical analyses. The Cox regression analysis was applied to calculate the connection between survival outcomes and gene expression. The log-rank test was used to calibrate the difference in the survival analysis with *p* < 0.05 indicated statistically significant.

### Supplementary information

Supplementary Figures S1

Table S1

Table S2

## Data Availability

The data that support the current work are available from GDC TCGA Breast Cancer (BRCA) (https://gdc.xenahubs.net) with dataset ID TCGA-BRCA.htseq_counts.tsv with a total of 1217 samples and extracted 127 samples of TNBC subtype of breast cancer for our analysis. The data used for validation in this article were obtained from METABRIC (Breast Cancer, Nature 2012 & Nat Commun 2016) (http://www.cbioportal.org/datasets) with a total of 2509 samples and we extracted 320 samples of TNBC subtype of breast cancer for our analysis and merged 320 samples with 127 TNBC sample obtained from TCGA-BRCA and used 447 patient data with TNBC as validation cohort, The data we have generated through this study can be found on figshare^53^. The stem cell expression profiles (syn2701943) were downloaded from the 91 Progenitor Cell Biology Consortium database (https://www.synapse.org)^54^ and the scRNA-seq dataset obtained from Gene Expression Omnibus (GEO) (https://www.ncbi.nlm.nih.gov/geo/) with the accession number GSE176078 (nTNBC = 10)^55^.

## Code availability

The analysis results associated with this paper is available on Github (https://github.com/saminagul12345/breast_cancer) and the R code used in the analysis of the data is available on Github (https://github.com/saminagul12345/breast_cancer).
