# Supplementary material for: Stemness signature and targeted therapeutic drugs identification for Triple Negative Breast Cancer
Source: Sci Data. 2023 Nov 20;10:815. doi: 10.1038/s41597-023-02709-8 (PMC10662149; doi:10.1038/s41597-023-02709-8)
Supplement: Supplementary file 1 — Supplementary Figures S1 [file 41597_2023_2709_MOESM1_ESM.pdf]

Contents

1. Supplementary Figure 1a, b
2. Supplementary Figure 2a-d
3. Supplementary Figure 3a,b
4. Supplementary Figure 4a,b
5. Supplementary Figure 5a
6. Supplementary Figure 6

Supplementary Figure 1a,b

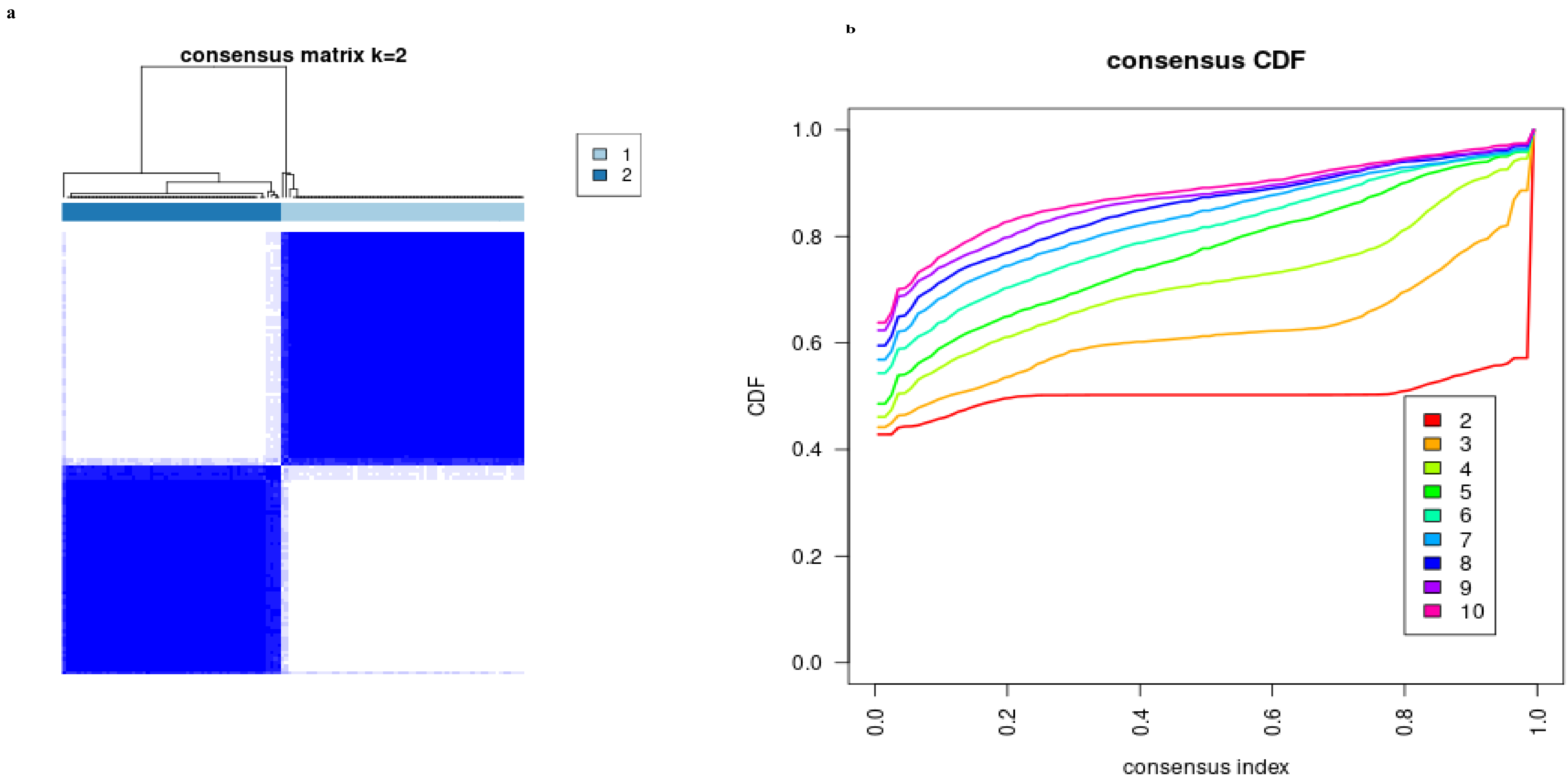

Figure 1. Identification of the two stemness subtypes. **(a)** Consensus clustering heatmap when k = 2. **(b)** CDF curves of the Consensus score from k = 2 to 10.

Supplementary Figure 2a-d

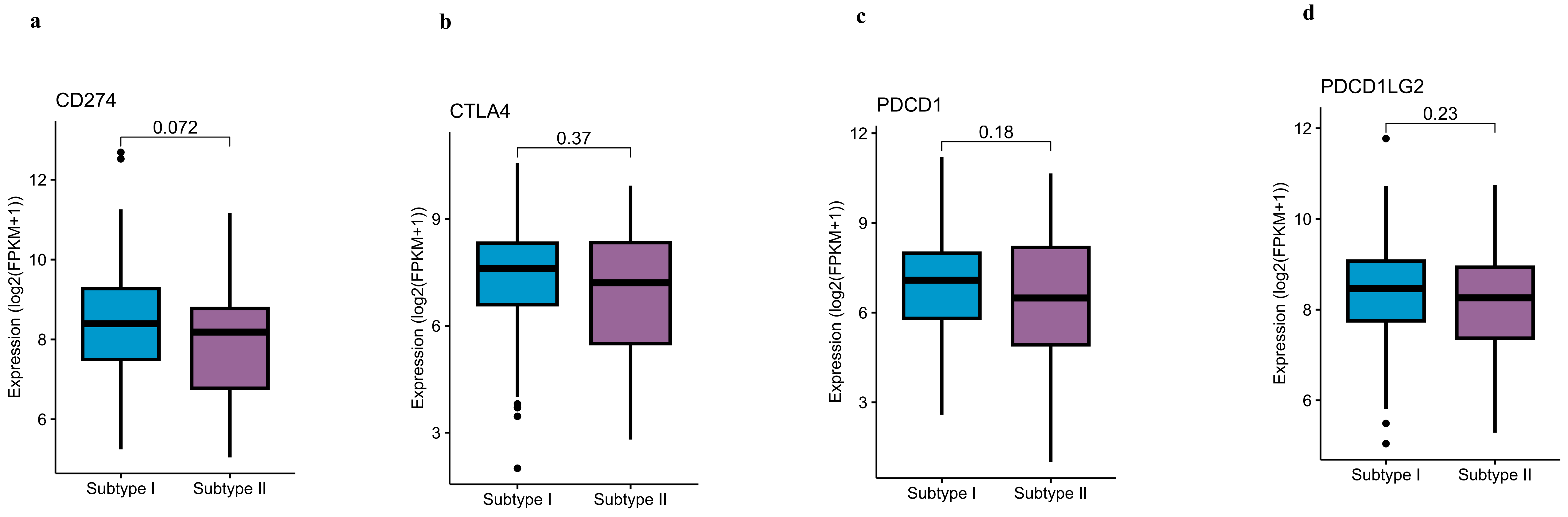

Figure 2. (a-d) The expression levels of CD274, CTLA4. PDCD1, PDCD1LG2 in stemness subtpe I and II

Supplementary Figure 3a,b

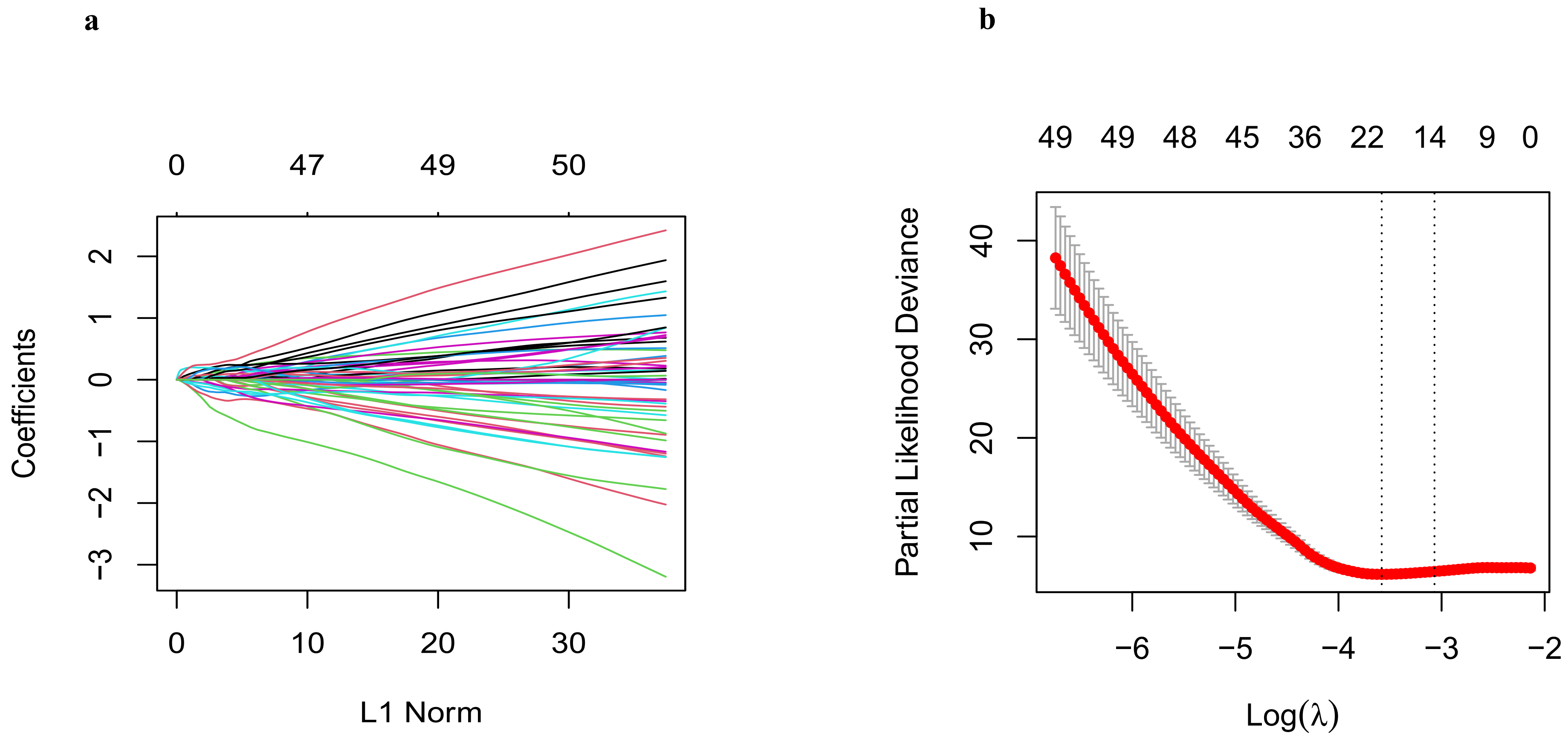

Figure 3. (a,b) Sixteen genes were identified by LASSO regression analysis

Supplementary Figure 4a,b

a

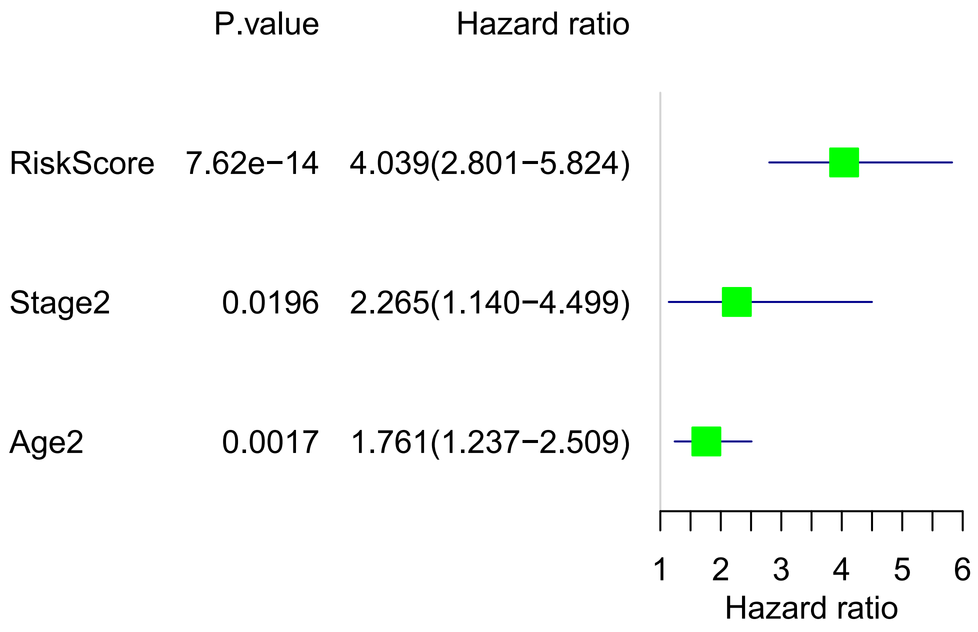

b

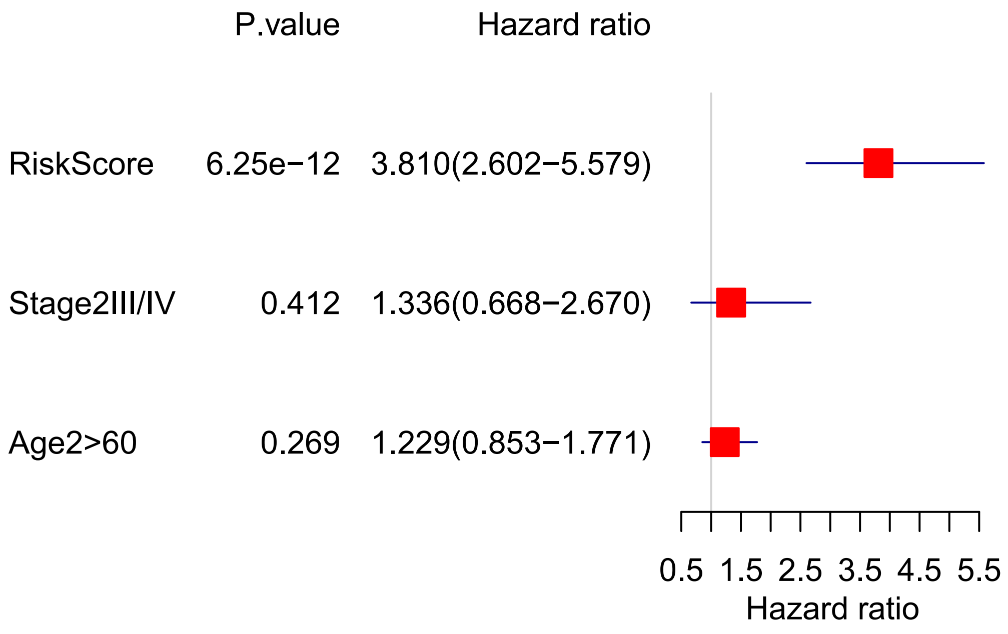

Figure 4. (a) The univariate Cox regression analysis. **(b)** The multivariate Cox regression analysis.

Supplementary Figure 5a

a

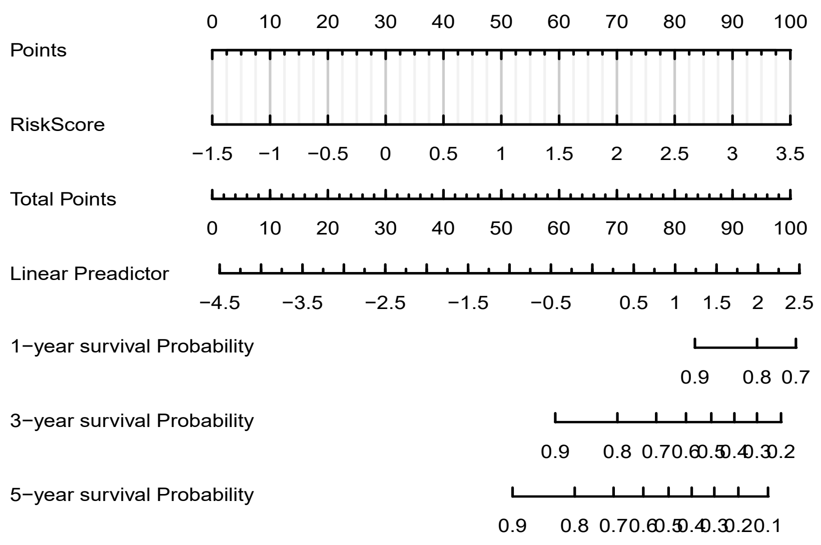

Figure 5. (a) Nomogram of the prediction model for TNBC

Supplementary Figure 6

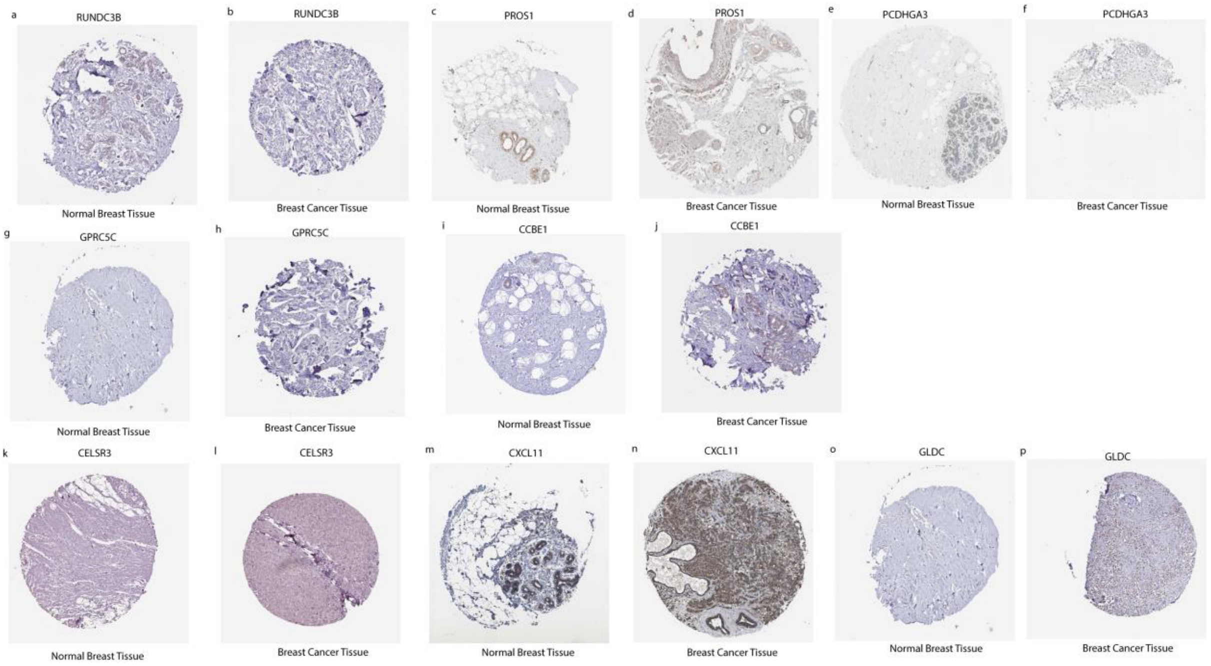

.**Figure 6.** The protein expression of the sixteen genes in Breast tumor tissues and normal tissues. The data were obtained from the HPA database. The expression profiles of the proteins encoded by RUNDC3B (**a, b**), PROS1 (**c, d**), PCDHGA3 (**e, f**), GPRC5C (**g, h**), CCBE1 (**i, j**), CELSR3 (**k, l**), CXCL11 (**m, n**), GLDC (**o, p**), p value < 0.05 was controlled, eight genes were not found in the database.
